# Altered association between cortisol and adrenocorticotropic hormone levels in the early stage of type 2 diabetic ketoacidosis

**DOI:** 10.3389/fendo.2025.1418357

**Published:** 2025-01-31

**Authors:** Liang Wang, Xiaomei Meng, Yuxiao Tang, Yaping Hao

**Affiliations:** Department of Endocrinology, The Affiliated Yantai Yuhuangding Hospital of Qingdao University, Yantai, China

**Keywords:** diabetes ketoacidosis, infection, bicarbonate, cortisol, altered cortisol ACTH association

## Abstract

**Objectives:**

In the early stages of various critical infections and diseases, altered association of cortisol and adrenocorticotropic hormone (ACTH) levels occurs, with cortisol levels increasing and ACTH levels remaining normal or decreasing. This study aimed to explore the relationship between ACTH and cortisol levels in patients with diabetic ketoacidosis (DKA) and the influence of the severity of DKA.

**Methods:**

A total of 106 type 2 diabetes patients with DKA admitted to the Endocrinology Department of Yantai Yuhuangding Hospital from February 2018 to May 2023 were divided into groups without (n=54) and with bacterial infection (n=52). Twenty type 2 diabetes patients without infection or DKA admitted during the same period were included as the control group. Cortisol and ACTH levels were measured on the first day of admission and the day after DKA correction for patients with DKA and on the first day of admission and the day before discharge for the control group.

**Results:**

Compared with the control group, the DKA groups both with and without infection had significantly higher cortisol levels (*P*<0.05) and significantly lower ACTH levels (*P*<0.01) at admission. DKA patients with infection had significantly higher cortisol levels at admission than those without infection (734.51 ± 348.69 nmol/L vs 508.79 ± 268.72 nmol/L, *P*<0.01), while ACTH levels did not differ significantly between the two groups (*P*>0.05). After correction of DKA, no differences in cortisol or ACTH levels were observed among the three groups. Compared with levels at admission, DKA patients both with and without infection had lower cortisol levels and higher ACTH levels after DKA correction (all *P*<0.001). Multiple stepwise regression analysis showed that for all DKA patients and for subgroups with and without infection, the cortisol level at admission was independently positively correlated with the ACTH level and negatively correlated with the bicarbonate level (both *P*<0.01).

**Conclusions:**

In the early stage of DKA, a phenomenon of altered association between cortisol–ACTH occurs and is especially prominent in DKA patients with infection. This altered association between cortisol–ACTH disappears after DKA correction, and the severity of DKA is an independent influencing factor on the cortisol level in early-stage DKA.

## 1 Introduction

Diabetic ketoacidosis (DKA) is a clinical syndrome of hyperglycemia, ketosis, and anion gap metabolic acidosis caused by abnormal metabolism of glucose, fat, and protein due to insufficient insulin levels or an increased level of insulin antagonist hormone. DKA remains one of the most frequently occurring diabetes-related emergencies, and despite advances in diabetes management and increased standardization of care, is still associated with appreciable morbidity and mortality (1, 2). Research has confirmed that infections, stress, and trauma are important triggers for DKA (2, 3). Cortisol is an important stress hormone that is secreted by the adrenal cortex and regulated by the hypothalamic pituitary adrenal (HPA) axis (4, 5). Under stress conditions, the HPA axis is activated, leading to increases in the levels of both cortisol and adrenocorticotropic hormone (ACTH), and this activation represents an important mechanism for adapting to stress and maintaining a stable internal environment. Previous studies have illustrated a close correlation between elevated cortisol levels and the severity of disease in multiple illness and stress states (6–8). However, an increasing number of animal and human studies in recent years have shown that in the early stages of critical illness, infections, and other diseases, altered association of cortisol and ACTH levels occurs that is characterized by increased cortisol levels and normal or decreased ACTH levels. Research related to this phenomenon has mainly focused on individuals hospitalized in an intensive care unit (ICU) and experiencing sepsis and/or shock (9–14). Changes in serum cortisol and ACTH levels during the onset of DKA have been rarely investigated.

The present study aimed to investigate the changes in cortisol and ACTH levels in patients with DKA as well as the potential mechanisms of these changes. The results provide important supplementary and reference value for the clinical diagnosis and treatment of DKA and further in-depth research on the phenomenon of altered cortisol–ACTH association.

## 2 Materials and methods

### 2.1 Study population and design

A total of 106 type 2 diabete mellitus (T2DM) patients with DKA admitted to the Endocrinology Department of Yantai Yuhuangding Hospital from February 2018 to May 2023 were included. The inclusion criteria were as follows: 1) diagnosed with DKA (15) based on blood glucose >11 mmol/L with a positive result for blood ketone or urine ketone; low blood pH (<7.3) or low blood bicarbonate (<15 mmol/L); 2) aged 18–75 years; and 3) no history of treatment for DKA before admission. The exclusion criteria included: 1) presence of another acute complication of diabetes; 2) diagnosis of gestational diabetes or another special type of diabetes; 3) conditions affecting HPA axis function, including adrenal cortical insufficiency or Cushing’s syndrome; severe liver or kidney dysfunction; treatment with exogenous glucocorticoids or any drug affecting the HPA axis; mental disorders or central nervous system diseases; 4) autoimmune diseases; and 5) malignant tumors.

All DKA patients were divided into two groups without bacterial infection (n=54) and with bacterial infection (n=52) based on the presence or absence of bacterial infection. The evaluation of bacterial infection included: 1) evidence of infection detected through analysis of blood, body fluids, or auxiliary examinations; 2) each patient’s clinical manifestations, routine blood test results, C-reactive protein level, and procalcitonin level. Twenty T2DM patients without infection or DKA admitted during the same time period served as the control group. This study was approved by the Ethics Committee of Yantai Yuhuangding Hospital. All patients provided written informed consent before participation.

### 2.2 Data collection and laboratory measurements

Venous blood was collected at admission for DKA before the start of treatment to measure blood glucose and bicarbonate levels. On the morning after admission, fasting blood samples were collected for measurement of albumin, blood lipid profile [total cholesterol (TC), triglycerides (TG), high-density lipoprotein cholesterol (HDL-c), low-density lipoprotein cholesterol (LDL-c)], glycated hemoglobin (HbA1c), alanine aminotransferase (ALT), and serum creatinine. Venous blood was collected for electro-chemiluminescence to determine cortisol and ACTH levels at 8:00 am on the day after admission and 8:00 am on the day after DKA correction (Cobas e601; Roche, Switzerland) for all patients with DKA and at 8:00 am on the day after admission and 8:00 am on the day before discharge for the control group. Body mass index was calculated as follows: BMI = weight (kilogram)/height^2^ (m^2^).

### 2.3 Statistical analyses

Statistical analyses were performed using SPSS 16.0. Data for all variables were tested for normality. Skewed distributed variables are represented as median (interquartile range), and normally distributed variables are represented as mean ± standard deviation (SD). Student’s *t*–test or analysis of variance was performed to compare data between or among groups for variables with a normal distribution, whereas the Mann-Whitney U-test or Kruskal–Wallis test was used to compare variables with a skewed distribution. The χ^2^ test was applied to detect differences in categorical variables among the groups. Comparison of cortisol and ACTH levels before and after DKA correction was performed using the independent sample t-test and two related sample tests. Multiple stepwise regression analysis was conducted to identify the factors that influenced the serum cortisol level.

## 3 Results

### 3.1 Clinical characteristics of patients

A total of 20 T2DM patients without DKA and 106 T2DM patients with DKA were included in this study. As shown in **Table 1**, compared with the control group, patients with DKA had higher blood glucose and lower levels of age and bicarbonate (all *P*<0.05). No significant differences were observed in gender, BMI, systolic blood pressure (SBP), diastolic blood pressure (DBP), HbA1c, albumin, TC, TG, LDL-c, HDL-c, ALT, and serum creatine (all *P*>0.05). In addition, compared with the DKA without infection group, the DKA with infection group had lower levels of bicarbonate (6.78 ± 3.73 mEq/L vs 9.45 ± 4.35 mEq/L, *P*=0.001) and albumin (33.03 ± 4.87 g/L vs 36.22 ± 4.82 g/L, *P*=0.001, while gender, age, SBP, DBP, blood glucose, HbA1c, TC, TG, LDL-c, HDL-c, ALT, and blood creatinine showed no statistically significant differences between the two groups (all *P*>0.05).

**Table 1:** Clinical characteristics of patients.

Characteristic	Control group (n=20)	DKA group (n=106)	*P*	DKA without infection (n=54)	DKA with infection (n=52)	*P*
Gender (male/female)	10/10	45/61	0.625	25/29	20/32	0.438
Age (years)	57.50(35.25-67.50)	35.50(26.50-58.00)	0.002	35 (24.75-57.25)	37 (27-58)	0.595
BMI (kg/m^2^)	26.86 ± 4.73	24.93 ± 5.34	0.537	25.51 ± 4.88	24.34 ± 5.65	0.257
SBP (mmHg)	136.70 ± 13.52	134.06 ± 17.71	0.101	137 ± 18	130 ± 16	0.059
DBP (mmHg)	78.8 ± 8.76	82.23 ± 13.02	0.065	82 ± 11	81 ± 14	0.725
HCO3 (mEq/L)	23.39 ± 1.73	8.14 ± 4.25	<0.001	9.45 ± 4.35	6.78 ± 3.73	0.001
Glucose (mmol/L)	15.79 ± 6.37	26.13 ± 10.44	0.045	27.58 + 10.06	24.63 ± 10.71	0.148
HbA1c (%)	10.26 ± 2.59	11.80 ± 2.72	0.429	11.70 + 2.89	11.90 + 2.54	0.709
Albumin (g/L)	38.49 ± 4.57	34.66 ± 5.08	0.667	36.22 ± 4.82	33.03 ± 4.87	0.001
TC (mmol/L)	4.67(3.93-5.81)	5.08(4.27-6.66)	0.134	5.43 (4.39-6.77)	4.85 (4.12-6.26)	0.083
TG (mmol/L)	2.02(1.13-3.63)	1.33(0.99-2.93)	0.431	1.38 (1.01-3.78)	1.28 (0.98-2.26)	0.342
LDL-c (mmol/L)	2.84 ± 1.15	3.00 ± 1.34	0.348	3.06 ± 1.40	2.93 ± 1.29	0.599
HDL-c (mmol/L)	1.20 ± 0.26	1.26 ± 0.45	0.358	1.26 ± 0.36	1.25 ± 0.53	0.934
ALT (U/L)	20.50(12.25-33.25)	15.00(10.00-23.00)	0.134	15 (11-22.25)	14 (9-24.5)	0.633
Serum creatine (µmmol/L)	51.00(46.50-68.5)	45.00(36.75-60.00)	0.057	47 (39.75-63.5)	42 (35.25-55.75)	0.144

### 3.2 Comparisons of cortisol and ACTH levels between different groups and at different times in the course of DKA

**Figure 1:**
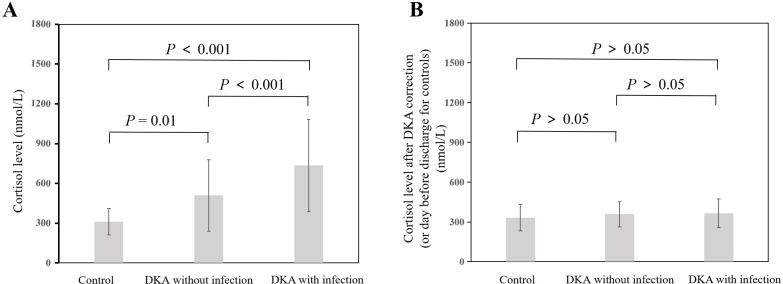
Comparison of cortisol levels among groups and at different time points. **(A)** Cortisol at admission; **(B)** Cortisol after correction of DKA (or day before discharge for controls).

**Figure 2:**
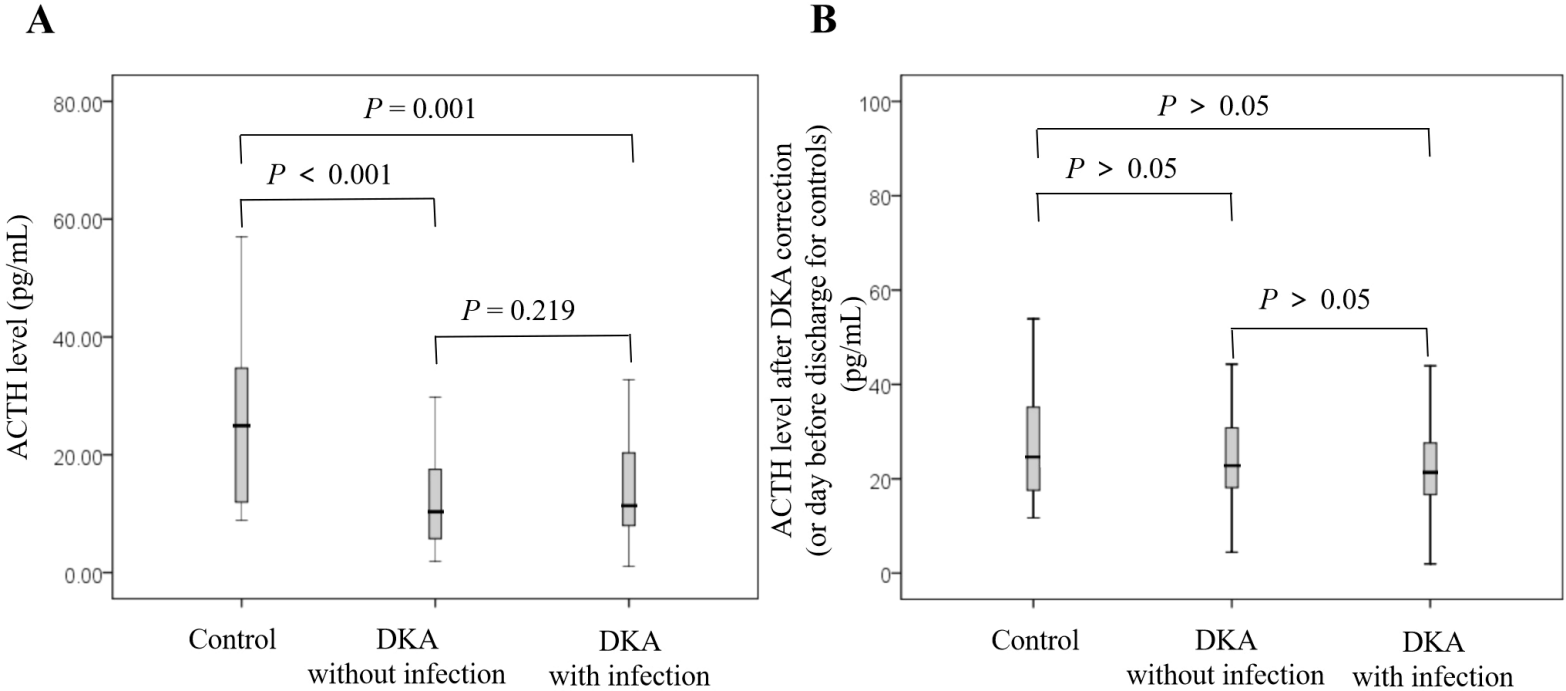
Comparison of ACTH levels among groups and at different time points. **(A)** ACTH at admission; **(B)** ACTH after correction of DKA (or day before discharge for controls).

As shown in **Figure 1A**, the cortisol level in the DKA groups both with and without infection was significantly higher than that in the control group at admission (all *P*<0.05), and the cortisol level in the DKA with infection group was significantly higher than that in the DKA without infection group at admission (734.51 ± 348.69 nmol/L vs 508.79 ± 268.72 nmol/L, *P*<0.001). The cortisol levels after DKA correction in DKA patients and on the day before discharge in the control group showed no significant difference among the three groups (all *P*>0.05, **Figure 1B**). As shown in **Figure 2A**, the ACTH level in the DKA groups both with and without infection was significantly lower than that in the control group at admission (all *P*<0.01), while the ACTH levels did not differ between the two DKA groups (*P*>0.05). As shown in **Figure 2B**, no difference in ACTH levels was observed after DKA correction (DKA patients) or the day before discharge (control group) among the three groups (all *P*>0.05).

Compared with the levels at admission, the DKA groups both with and without infection showed a decrease in cortisol level and an increase in ACTH level after DKA correction (all *P*<0.001; **Table 2**).

**Table 2:** Cortisol and ACTH levels among different groups and at different times.

Characteristic	Cortisol (at admission), nmol/L	Cortisol (after DKA correction), nmol/L	ACTH (at admission), pg/mL	ACTH (after DKA correction), pg/mL
Control group	311.27 ± 100.15	330.60 ± 99.18^a^	24.94(11.83-36.08)	24.62(17.37-25.59) ^a^
DKA without infection	508.79 ± 268.72	357.51 ± 94.47^#^	10.33(5.57-17.73)	22.80(17.95-31.45)^*^
DKA with infection	734.51 ± 348.69	364.23 ± 109.03^#^	11.35(7.92-20.68)	21.33(16.6-27.65)^*^

a Cortisol and ACTH were detected on the day before the discharge for the control group.

^#^ *P<*0.001 compared with cortisol (at admission), *P<0.001 compared with ACTH (at admission).

### 3.3 Independent influencing factors for cortisol levels in early-stage DKA

For the multiple stepwise regression analysis, cortisol level was set as the dependent variable, and the independent variables included age, gender, BMI, HbA1c, blood glucose at admission, ACTH at admission, bicarbonate, albumin, blood lipid levels, ALT, blood creatinine, and presence of infection. Multiple stepwise regression showed that the cortisol level at admission was independently positively correlated with the ACTH level at admission (standardized β=0.354, *P*<0.001), the presence of infection (standardized β=0.226, *P*=0.005), DBP (standardized β=0.219, *P*=0.003), and blood glucose at admission (standardized β=0.208, *P*=0.006), and independently negatively correlated with the bicarbonate level (standardized β=-0.341, *P*<0.001).

Further subgroup analysis showed that the cortisol level at admission was positively correlated with the ACTH level at admission (DKA without infection group, standardized β=0.286, *P*=0.003; DKA with infection group, standardization β=0.337, *P*=0.002) and independently negatively correlated with the bicarbonate level (DKA without infection group, standardized β=-0.367, *P*<0.001; DKA with infection group, standardization β=-0.489, *P*<0.001). In the DKA without infection group, the cortisol level at admission was independently positively correlated with the creatinine level at admission. In the DKA with infection group, the cortisol level at admission was independently positively correlated with DBP, ALT, and blood glucose at admission (all *P*<0.05; **Table 3**).

**Table 3:** Factors influencing the cortisol level at admission in DKA patients.

	Standardized β	t	*P*
All DKA patients			
ACTH	0.354	4.783	<0.001
Bicarbonate	-0.341	-4.391	<0.001
DBP	0.219	2.995	0.003
Blood glucose (at admission)	0.208	2.786	0.006
Infection	0.226	2.873	0.005
DKA patients without infection			
Serum creatine	0.527	5.76	<0.001
ACTH	0.286	3.12	0.003
Bicarbonate	-0.367	-4.125	<0.001
DKA patients with infection			
ACTH	0.337	3.349	0.002
Bicarbonate	-0.489	-4.544	<0.001
DBP	0.379	3.702	0.001
ALT	0.398	2.818	0.007
Blood glucose (at admission)	0.210	2.082	0.043

## 4 Discussion

The results of the present study indicate that in the early stage of DKA, the phenomenon of altered cortisol–ACTH association exists and is especially prominent in DKA patients with bacterial infection. The altered association of cortisol and ACTH levels disappeared after correction of DKA in these patients. Also, the severity of DKA, as represented by the bicarbonate level, was an independent influencing factor for the cortisol level in the early stage of DKA.

The study of the adrenal cortex axis in patients with critical illness has always received widespread attention, and patients included in previous studies tended to have hypercortisolemia, which is directly proportional to the severity of disease (16, 17). The present study found that in cases both with and without bacterial infection, the cortisol level was significantly increased in the early stages of DKA, and the cortisol level increased with increasing severity of DKA (represented by a greater decrease in bicarbonate), consistent with previous research results in patients with critical illness. It has been widely recognized in previous studies that high plasma cortisol levels in patients with critical illness are driven by increased synthesis of corticotropin-releasing hormone, ACTH, and arginine vasopressin (6, 18), and our study also found that the cortisol level increased with increasing ACTH level in the early stage of DKA. In recent years, numerous studies have reported an altered association of ACTH and cortisol levels in stress disease states of non-critical diseases other than sepsis (19), including chronic obstructive pulmonary disease, the inflammatory response, and psychiatric disorders (12, 20–22), suggesting the existence of another mechanism for the maintenance of high glucocorticoid levels in addition to ACTH.

As early as 1995, Vermes et al. (19) found that in critically ill patients, the ACTH level only briefly increases, while the cortisol level remains consistently high. Boonen et al. (14) reported that among patients admitted to the ICU, the plasma ACTH level remained consistently low from day 1 to week 1 after admission, while the plasma total cortisol and free cortisol levels remained high over this period. Another study on burn patients found that the plasma cortisol concentration increased proportionally to the burn area, while the plasma ACTH level did not increase (13). In patients undergoing cardiac surgery, plasma ACTH and cortisol levels begin to rise shortly after surgery, but ACTH quickly decreases while plasma cortisol can remain high (23). A recent meta-analysis on coronavirus disease 2019 (COVID-19), including 440 patients with COVID-19 and 474 control subjects, found significantly higher cortisol levels in COVID-19 patients compared with controls, but no significant difference in ACTH levels (24). In addition, the phenomenon of cortisol–ACTH dissociation was observed in a mouse model of sepsis (25). The present study also observed the cortisol–ACTH altered association in the early stages of DKA, and this cortisol-ACTH altered association disappeared after the correction of DKA.

The mechanism of altered cortisol-ACTH association remains unclear. Some studies have shown that increased production and decreased decomposition of cortisol in critically ill patients leads to hypercortisolemia. Marik et al. (10) found that patients with systemic inflammatory response syndrome (SIRS) have an increased rate of cortisol production while the ACTH level is decreased. They also reported a lower degree of cortisol response under exogenous ACTH stimulation, while pro-inflammatory factors tumor necrosis factor alpha and interleukin 6 show a positive correlation with cortisol production, suggesting that these cytokines may be driving factors for increased cortisol production during critical illness. Some studies also found that elevated cortisol levels in patients with critical illness may be the result of reduced cortisol clearance. The liver and kidney are the main sites for cortisol clearance. A-ring reductase (5β-reductase and 5α-reductase) and 11 β-hydroxysteroid dehydrogenase type 2 (11β-HSD2) play an important role in cortisol metabolism (26, 27). Boonen et al. (14) confirmed that the increase in blood cortisol in patients with critical illness is mainly caused by a decrease in cortisol clearance rather than an increase in cortisol production and confirmed that the decrease in cortisol clearance is related to lower expression and reduced activity of A-ring reductase and 11β-HSD2. The present study also found that serum creatinine and ALT levels were independent factors positively correlated to the increased cortisol level in patients with DKA. We speculate that the increase in cortisol during DKA is partially caused by abnormal liver and kidney function leading to a decrease in cortisol clearance.

Although an altered cortisol-ACTH association has been commonly observed in patients with critical illness, research has suggested that only the plasma cortisol level is associated with disease severity, while the ACTH level is not associated with disease severity (13). In the present study, an independent association was also found between the high cortisol level and low bicarbonate level in DKA patients.

The present study has several limitations to consider. First, the sample size was relatively small for drawing robust conclusions. Second, the single-center design may have resulted in selection bias. In our future research, we will expand the sample size and conduct a mechanistic study to validate the conclusions of this study.

In conclusion, the results of this study provide evidence that in the early stage of DKA, a phenomenon of altered cortisol–ACTH association exists, which then disappears after correction of DKA. The severity of DKA (as represented by the bicarbonate level) is an independent influencing factor for the cortisol level in the early stage of DKA. Compared to other critical illnesses, DKA has fewer influencing factors. Therefore, these findings are of great significance for the in-depth study of the mechanism of altered cortisol-ACTH association.

## Data Availability

The raw data supporting the conclusions of this article will be made available by the authors, without undue reservation.
